# Heterologous, Expression, and Characterization of Thermostable Glucoamylase Derived from* Aspergillus flavus* NSH9 in* Pichia pastoris*

**DOI:** 10.1155/2016/5962028

**Published:** 2016-07-18

**Authors:** Kazi Muhammad Rezaul Karim, Ahmad Husaini, Md. Anowar Hossain, Ngieng Ngui Sing, Fazia Mohd Sinang, Mohd Hasnain Md. Hussain, Hairul Azman Roslan

**Affiliations:** ^1^Department of Molecular Biology, Faculty of Resource Science and Technology, Universiti Malaysia Sarawak, 94300 Kota Samarahan, Sarawak, Malaysia; ^2^Institute of Nutrition and Food Science, University of Dhaka, Dhaka 1000, Bangladesh; ^3^Department of Biochemistry and Molecular Biology, University of Rajshahi, Rajshahi 6205, Bangladesh

## Abstract

A novel thermostable glucoamylase cDNA without starch binding domain (SBD) of* Aspergillus flavus* NSH9 was successfully identified, isolated, and overexpressed in* Pichia pastoris* GS115. The complete open reading frame of glucoamylase from* Aspergillus flavus* NSH9 was identified by employing PCR that encodes 493 amino acids lacking in the SBD. The first 17 amino acids were presumed to be a signal peptide. The cDNA was cloned into* Pichia pastoris* and the highest expression of recombinant glucoamylase (rGA) was observed after 8 days of incubation period with 1% methanol. The molecular weight of the purified rGA was about 78 kDa and exhibited optimum catalytic activity at pH 5.0 and temperature of 70°C. The enzyme was stable at higher temperature with 50% of residual activity observed after 20 min at 90°C and 100°C. Low concentration of metal (Mg^++^, Fe^++^, Zn^++^, Cu^++^, and Pb^++^) had positive effect on rGA activity. This rGA has the potential for use and application in the saccharification steps, due to its thermostability, in the starch processing industries.

## 1. Introduction

Glucoamylase (1,4-*α*-D-glucan glucohydrolase, EC 3.2.1.3, GA) is an exoacting enzyme that yields *β*-D-glucose from the nonreducing ends of starch and related oligo- and polysaccharide chains by hydrolyzing *α*-1,4 and *α*-1,6 linkages [1]. This enzyme is able to completely hydrolyze starch if incubated for extended periods of time and hence called the saccharifying enzyme. Glucoamylase is an important group of enzymes in starch processing in the food industries, as it is used for the production of glucose and fructose syrup from liquefied starch [2]. It is also used in baking, juice, beverage, pharmaceuticals, confectionery, and many fermented foodstuffs industries for commercial production [3]. It is the most important type of industrial enzyme because of its widespread uses together with *α*-amylases and debranching enzymes, in the saccharification of starch to yield soluble sugars, which are widely used by many food industries and in the production of bioethanol [4].

From a structural point of view, glucoamylase enzymes constitute the bulk of family GH15 of glycoside hydrolases [5]. Glucoamylase (GA) can be derived mostly in microorganisms and also in animals and plants. A large number of microbes, including bacteria, yeast, and fungi are capable of producing glucoamylase. Filamentous fungi constitute the major source of all microorganisms. However, the exclusive productions of glucoamylase in the enzyme industry are achieved by* Aspergillus niger* [6],* Aspergillus oryzae*, and* Rhizopus oryzae* [7].

Fungi often produce more than one form of enzyme that may have different molecular weights, amino acid composition, glycosylation, ability to digest soluble and raw starches, and stability [8]. The majority of fungal glucoamylase is multidomain consisting of a catalytic domain at the N-terminus and a starch binding domain (SBD) at the C-terminus [9], which is separated by an O-glycosylated linker region rich in serine and threonine residues. But with exception, glucoamylases with a single domain structure are also available in* A. niger* [10],* Rhizopus oryzae* [11], and* A. oryzae *[12]. Two different forms of glucoamylase, with and without starch binding domain, are produced from one gene or two different genes. For example,* A. niger* and* A. awamori* var.* kawachi* produce two forms of glucoamylase (GA) from the same gene [13], while* A. oryzae* produces two GA from two different genes [14].

Due to industrial importance, glucoamylase encoding gene has been cloned, characterized, and heterologously expressed which derived the* Aspergillus *species,* A. niger* [15],* A. awamori *var.* kawachi *[16], and* A. oryzae* [12]. El-Abyad and his coworker [17] screened 21 species for amylolytic activity and found that* A. flavus* was among the most active amylase producer. It is also reported that* A. flavus* NSH9 was the best amylase producer among fourteen isolates of* Aspergillus* sp. [18]. Although glucoamylase from* A. flavus* has been characterized [18, 19], little is known about its molecular information. The genetic sequence, molecular information, substrate specificity, and biochemical characteristic of glucoamylase from* A. flavus *are not yet well known. On the other hand,* Pichia pastoris* is one of the most used eukaryotic systems for the production of recombinant proteins. It is easy to culture and can reach high cell densities and has strong and regulated promoters and ability to secrete proteins as well as introducing posttranslational modifications [20, 21]. Here, we described the isolation, cloning, and sequence analysis of glucoamylase gene (GA) derived from* Aspergillus flavus* and its expression in* Pichia pastoris*.

## 2. Materials and Methods

### 2.1. Maintenance and Culturing of* Aspergillus* Strain

A newly fungal strain of* Aspergillus flavus* NSH9 with starch degrading properties was previously isolated from the sago humus and kept as collection at the Department of Molecular Biology, Faculty of Resource Science and Technology, Universiti Malaysia Sarawak, Malaysia. For enzyme induction, the actively growing fungal mycelium was transferred from potato dextrose agar (PDA) plate to a culture medium containing (in g L^−1^) 20 g raw sago starch, 3 g KH_2_PO_4_, 1 g (NH_4_)_2_SO_4_, 0.5 g MgSO_4_·7H_2_O, and 4 g of yeast extract. The mycelia were collected after 4 days of growth in the culture media at room temperature of 28°C with shaking at 150 rpm and then subsequently used to extract the total RNA.

### 2.2. Extraction of Total RNA and DNA and First-Strand cDNA Synthesis

Total RNA was extracted by using TRIzol Reagent according to the protocol as described by Schumann et al. [22] and the genomic DNA was extracted according to the method of Cubero et al. [23]. Total RNA was purified by being treated with DNAse and reverse transcriptions were carried out in 20 *μ*L reaction mixture containing the following: 0.1–5 *μ*g DNA free RNA as a template, 0.5 *μ*g oligo (dt)_18_, 4 *μ*L of 5x reaction buffer, 2 *μ*L 10 mM dNTP Mix, 20 U Riblock RNase Inhibitor, and 40 U Moloney Murine Leukemia Virus (M-MuLV) reverse transcriptase (Thermo Scientific). The reaction was performed at 37°C for 60 min and at 70°C for 5 min. The product was either used immediately in the subsequent PCR analysis or stored at −80°C.

### 2.3. Isolation and Subcloning of Glucoamylase cDNA

Glucoamylase gene from* Aspergillus flavus* NSH9 was isolated by using GAAsp-F and GAAsp-R primers that were designed based on sequences of* A. flavus *NRRL3357 (glucoamylase precursor putative mRNA, XM_002374964.1) obtained from the National Center for Biotechnology Information (NCBI). The PCR was carried out in 50 *μ*L reaction mixture containing the following: 5 *μ*L 10x Pol Buffer A (1x final concentration), 5 mM MgCl_2_, 0.2 mM dNTP, 0.1–0.5 *μ*M each of the forward and reverse gene specific primers, 0.5 *μ*g cDNA template, and 1.25 U* Taq* DNA polymerase (EURx, Gdansk Poland). The full length of* A. flavus* NSH9 glucoamylase cDNA and glucoamylase gene were amplified by PCR, using the following oligonucleotide primers: GAAsp_F (5′-CGCATGCGGAACAACCTTCTTT-3′) and GAAsp_R (5′-CTACCACGACCCAACAGTTGG-3′). The PCR reaction was carried out using the following conditions: denaturation at 94°C for 2 min for one cycle, followed by denaturation at 94°C for 45 sec, annealing at 55°C for 45 sec, and elongation at 72°C for 1.45 min for 35 cycles, and a final elongation was at 72°C for 5 min. The purified PCR product was then cloned into pGEMT-Easy Vector (Promega) as described by the manufacturer protocol and transformed into* E. coli* XL1-Blue. Purified plasmid was run on agarose gel and sequenced using T7 and SP6 primers.

### 2.4. Cloning of Glucoamylase cDNA into Yeast Expression Vector

The pPICZ*α*C (Invitrogen) was selected as expression vector and used for the cloning of glucoamylase cDNA without signal peptide sequences. A putative signal sequence was predicted by the Signal-3L program [24], and glucoamylase cDNA without the signal peptide was amplified using the following list of primers: forward primer (5′-AGC**GAATTC**A*GATTACAAG GACGACGACGATAAG* CCGTCCTTCCCTATCCAT-3′) and reverse primer (5′-ACGAC**TCTAGA**GCCCACGACCCAACAGTTGG-3′). The designed primers contained an* Eco*R1 site and* Xba*l site in the forward and reverse primers (bold and underline), respectively. In addition, the forward primer was designed to include FLAG tag containing 24 nucleotides (DYKDDDDK) and the reverse primer lacking in the native stop codon. The primers were designed to be in frame with the C-terminal 6x His tag located in the pPICZ*α*C. PCR was performed as follows: 1 cycle at 94°C for 2 min followed by 35 cycles of the sequence at 94°C for 45 sec, 62°C for 45 sec and 72°C for 1.45 min, and final extension 72°C for 5 min. After purification, the PCR product was digested with* Eco*R1 and* Xba*1 and ligated into pPICZ*α*C, producing the* P. pastoris* expression plasmid pPICZ*α*C_GA. The recombinant expression plasmid containing glucoamylase cDNA (pPICZ*α*C_GA) was then transformed into* Escherichia coli *XL1-Blue. The insert in pPICZ*α*C_GA was confirmed by PCR, restriction enzyme digestion, and sequencing analysis for correct orientation.

### 2.5. Transformation and Selection of Glucoamylase Transformant

The pPICZ*α*C_GA carrying the insert was linearized by* Sac*I and transformed into* P. pastoris* GS115 (Invitrogen) competent cells. The competent cells were prepared by using the Pichia EasyComp Transformation kit according to the instruction guidelines (Invitrogen). Linearized vector pPICZ*α*C, without insert, was also transformed into* P. pastoris* GS115 and used as a control. After transformation,* Pichia* cells were plated onto yeast extract peptone dextrose sorbitol (YPDS) agar plate containing 150 *μ*g mL^−1^ zeocin and incubated for 3 to 8 days at 30°C. Transformed colonies were confirmed by colony PCR using 5′*AOX1* and 3′*AOX1* primers and glucoamylase gene specific primers. Putative Mut^+^ colony was selected based on PCR using* AXO1* primers. Several Mut^+^ colonies from YPDS agar plate were selected and independently blotted onto Buffered Methanol-complex Medium (BMMY) (containing 1% (w/v) yeast extract, 2% (w/v) peptone, 1.34% (w/v) YNB, 4 × 10^−5^% (w/v) biotin, 0.5% (v/v) methanol, and 100 mM potassium phosphate buffer pH 6.0) agar plate containing 1% (w/v) soluble starch. Glucoamylase producing transformants were identified by the formation of decolorization zone or clear halo around the* Pichia* colonies after the addition of iodine solution.

### 2.6. Expression of Recombinant Glucoamylase (rGA)

The Mut^+^ GS115 single colony from YPDS agar plates was inoculated into 25 mL Buffered Glycerol-complex Medium ((BMGY) containing 1% (w/v) yeast extract, 2% (w/v) peptone, 1.34% (w/v) YNB, 4 × 10^−5^% (w/v) biotin, 1% (v/v) glycerol, and 100 mM potassium phosphate buffer pH 6.0) in 100 mL conical flask and incubated at 29°C with shaking of 230 rpm until the culture reached an OD_600_ of 2–4 (approximately after 24 hours). After that, cells were harvested by centrifugation at 3000 ×g for 5 min at room temperature, resuspended to an OD_600_ of 1.0 in Buffered Methanol-complex Medium ((BMMY) containing 1% (w/v) yeast extract, 2% (w/v) peptone, 1.34% (w/v) YNB, 4 × 10^−5^% (w/v) biotin, 0.5% (v/v) methanol, and 100 mM potassium phosphate buffer pH 6.0) (40 mL) in 250 mL conical flask, and incubated for 10 days at 29°C with shaking of 230 rpm. Approximately, 1 mL of the culture was withdrawn at every 24-hour intervals till 10-day period of incubation and added with absolute methanol into the culture to make the final concentration of 0.5% (v/v) of methanol. Based on the best production of recombinant glucoamylase, a* Pichia* cell was scaled up by increasing the methanol concentration to up to 5% (v/v) of final concentration.* Pichia pastoris* GS115 containing vector without the insert was also expressed as the above procedure and used as a control (Invitrogen 2010).

### 2.7. Electrophoresis of the Recombinant Glucoamylase

Nondenaturing PAGE and complete SDS-PAGE were both used to check on the glucoamylase enzyme activity and to estimate the molecular weight of the rGA expressed. Electrophoresis was carried out by using a 12% (w/v) acrylamide resolving gel and 6% (w/v) acrylamide stacking gel, as described by Laemmli [25] using the Mini-Protean III electrophoresis system (Bio-Rad, Richmond, CA, USA). The proteins were stained with Coomassie Brilliant Blue R-250. As for amylase activity staining of the recombinant glucoamylase (samples without heating and beta-mercaptoethanol on gel) was performed on a PAGE gel after washing the gel for 30 min with 0.1 M sodium acetate buffer (pH 5.0) containing 0.2% (v/v) Triton X-100 to remove the SDS in gel. After washing the gel was washed again with 0.1 M sodium acetate buffer (pH 5.0) containing 1% (w/v) soluble starch for 30 min. The recombinant glucoamylase activity appeared as clear band on dark blue background after the staining of iodine solution (0.15% (v/v) of iodine and 0.5% (w/v) of potassium iodide). Desired band in the complete SDS-PAGE analysis after the destaining from Coomassie Brilliant Blue R-250 was cut out and subjected to peptide sequencing. The analysis of the protein was done by MALDI-TOF/TOF mass spectrometry using a 5800 Proteomics Analyzer. The spectra generated were evaluated and analyzed to identify the protein of interest using Mascot sequence matching software (Matrix Science) with MSPnr100 Database. This analysis was done by Proteomics International Pty Ltd., Broadway, Nedlands, Western Australia.

### 2.8. Recombinant Glucoamylase Enzyme Activity and Protein Assay

Glucoamylase activity was determined by transferring a volume of 0.5 mL of the dilute glucoamylase enzyme followed by the addition of 0.5 mL of 1% (w/v) soluble starch solution in 0.1 M sodium acetate buffer (pH 5.0) and incubated at 55°C for 30 min. The released glucose was measured using 3,5-dinitrosalicylic acid (DNS) reagent with some modification [26] and measured at 540 nm. The enzyme activities were calculated using a calibration curve prepared using D-glucose as standard. One unit of glucoamylase activity was defined as the amount of enzyme that released 1 *μ*mol of glucose equivalent per minute from soluble starch under the assay condition. The protein content was determined by the method of Bradford [27] with bovine serum albumin as standard. The specific activity of recombinant glucoamylase was taken as unit of mg^−1^ protein.

### 2.9. Purification and Characterization of Recombinant Glucoamylase (rGA)

#### 2.9.1. Purification

Recombinant glucoamylase was purified by using an anti-FLAG M2 affinity gel according to the manufacturer's instruction (Sigma Aldrich).

#### 2.9.2. Effect of pH and Temperature

The optimum pH for activity was determined by measuring the glucoamylase activity at 55°C for 30 min using various types of buffers. The following 0.1 M buffer systems were used: sodium citrate (pH 3.0); sodium acetate (pH 4.0–5.0); potassium phosphate (pH 6.0–7.0); and Tris-HCI (pH 8.0–9.0). The optimum temperature for activity was assayed by measuring enzyme activity at optimum pH (0.1 M sodium acetate buffer, pH 5.0) over different temperature ranging from 20 to 90°C.

#### 2.9.3. pH Stability and Thermostability of rGA

For pH stability, the recombinant glucoamylase was dispersed (1 : 1) into 0.1 M buffer solution pH 3.0 to 9.0 and incubated at 25°C for 24 h [28] and after that an aliquot was used to determine the remaining activity at optimum pH and temperature. Thermal stability of the recombinant glucoamylase was determined by incubating in 0.1 M sodium acetate buffer pH 5.0, at 60–100°C for 90 min, and also enzyme was incubated in water at 70°C to observe the stability at neutral pH. Time course aliquots were withdrawn, cooled down in ice bath, and assayed under standard conditions at optimum pH (pH 5.0) and temperature (70°C).

#### 2.9.4. Effect of Substrate Concentrations

Enzyme activity was assayed in the reaction mixture containing different amount of soluble starch (0.25–2.0% (w/v)) at optimum pH 5.0 and temperature at 70°C with under standard assay conditions. The data were plotted (according to Lineweaver-Burk) to determine *V*_max_⁡ and *K*_m_ values.

#### 2.9.5. Effect of Metal Ions

In order to investigate the effect of metal ions (Na^+^, K^+^, Cu^2+^, Fe^2+^, Pb^2+^, Ca^2+^, Mg^2+^, Cd^2+^, and Zn^2+^) on the activity of recombinant glucoamylase, an enzyme aliquot was incubated with a metal ion solution (the final concentration of 1 mM and 5 mM and the final volume after addition substrate) for 30 minutes at 37°C. After that the remaining activity was measured under standard assay condition with optimum pH (pH 5.0) and temperature (70°C). The relative glucoamylase enzyme activity which was assayed in the absence of metal ions was taken as 1.00.

#### 2.9.6. Effect of Different Substrate

Different substrates such as soluble starch, amylopectin, glycogen, and gelatinized raw sago starch were used to investigate the hydrolyzing capacity of the recombinant glucoamylase produced.

#### 2.9.7. Bioinformatics

Nucleotide sequences of cDNA of glucoamylase were used as the query sequences in the BLAST searches using the BLAST X program. Searches of protein sequences were done in the NCBI (Protein BLAST). The phylogenic tree of glucoamylase from* Aspergillus flavus* NSH9 was constructed by using MEGA6 software [29]. For this the alignment of amino acid sequences was performed using the CLUSTALW program, and the evolutionary tree was calculated from the manually adjusted alignment with the neighbor-joining clustering method and the bootstrapping procedure. The number of bootstrap trails used was 1000 and implemented in the MEGA6 program.

## 3. Results

### 3.1. Identification and Analysis of Glucoamylase Gene

For isolation of glucoamylase from the cDNA and genomic DNA, total RNA and DNA were extracted from* Aspergillus flavus* NSH9 and mRNA was converted to cDNA. PCR amplified product by* GAAsp_F* (5′-CGCATGCGGAACAACCTTCTTT-3′) and* GAAsp_R* (5′-CTACCACGACCCAACAGTTGG-3′) primer indicated that the full length of glucoamylase was 1482 bp from cDNA and 1587 bp from DNA. A comparison of the genomic and cDNA sequences of glucoamylase indicated the presence of two introns varying in sizes of 59 bp and 46 bp. The open reading frame (ORF) of* A. flavus* NSH9 glucoamylase gene consisted of 1482 nucleotides, encoding 493 amino acid residues. The genes were submitted to GeneBank and assigned with their accession number as KU095082 and KU095083 for glucoamylase cDNA and genomic DNA, respectively. Based on the sequence comparison with other fungal glycosyl hydrolases, the glucoamylase protein can be assigned to glycosyl hydrolase family 15, and also in NCBI protein BLAST it was observed that this GA belonged to the glycoside hydrolases' 15 families lacking in starch binding domain (SBD). Five motifs, AEPKF, WGRPQRDGP, DLWEEV, ALSNHK, and AAELLYDA, were found highly conserved among fungi glucoamylase. The first 17 amino acids were presumed to be the signal peptide. Five putative asparagine-linked N-glycosylation sites (at 139, 198, 255, 369, and 457 position) and another two possible glycosylation sites (at 120 and 384), which were deduced according to the rule of Asn-X-Thr/Ser, were present in the deduced amino acid sequence. Searches of protein sequences in the NCBI (Protein BLAST) service revealed that the sequence of* A. flavus* NSH9 glucoamylase showed a high degree of identification with the glucoamylase sequences from other fungal glucoamylases.

**Figure 1:**
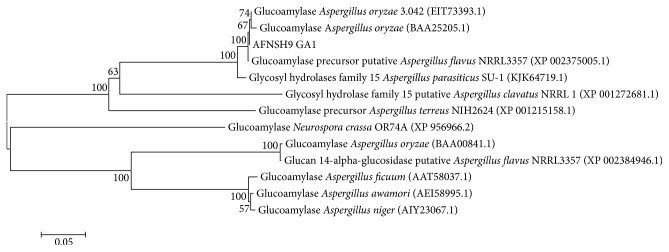
Homology tree between* Aspergillus flavus* NSH9 (AFNSH9 GA1) glucoamylase and other fungal glucoamylases based on amino acids sequences by MEGA6 software. Bar = 5 amino acid substitution per 100 amino acids.

The phylogenic tree of glucoamylase from* Aspergillus flavus* NSH9 was constructed by using MEGA6 software [29] (Figure 1). The results of BLAST between* A. flavus* NSH9 glucoamylase and other glucoamylases showed about 98-99% identical of* A. flavus* NRRL3357 (XP_002375005.1, putative precursor),* A. oryzae* 3.042 (EIT73393.1), and* A. parasiticus* SU1 (KJK64719.1). It was also found to be 70% identical with glucoamylase precursor of* A. terreus* NH2624 (XP_001215158.1). About 57% to 60% identical were also observed from* A. awamori* (AEI58995.1),* A. niger* (AIY23067.1),* A. ficuum* (AAT58037.1) and* Neurospora crassa* OR74A (EAA27730.2), and* A. oryzae* (BAA00841.1) and putative glucan 1,4 *α*-glucosidase was observed from* A. flavus* NRRL3357 (XP_002384946.1). In the phylogenic tree (Figure 1) and NCBI protein BLAST, it was found that glucoamylase from* A. flavus* NSH9 matched with the two types of glucoamylase from* A. oryzae* and* A. flavus* NRRL3357. It is assumed that* A. flavus* NSH9 also produced two glucoamylases, one that is lacking starch binding domain and the other with the starch binding domain.

The amino acid sequences of glucoamylase deduced from the nucleotide sequences were compared with those of other glucoamylases (gene accession numbers p69327 and p69328) retrieved from the Universal Protein Resources Knowledgebase (UniPort) and GeneBank [30] database. Based on the analysis of the data set, it is observed that the two amino acids, aspartic acid at the 203 position and glutamic acids at 206 position, were responsible for catalytic activity of glucoamylase of* A. flavus* NSH9 and tryptophan at 147 position was responsible for the binding.

### 3.2. Transformation, Expression of Glucoamylase cDNA, and Purification of rGA

**Figure 2:**
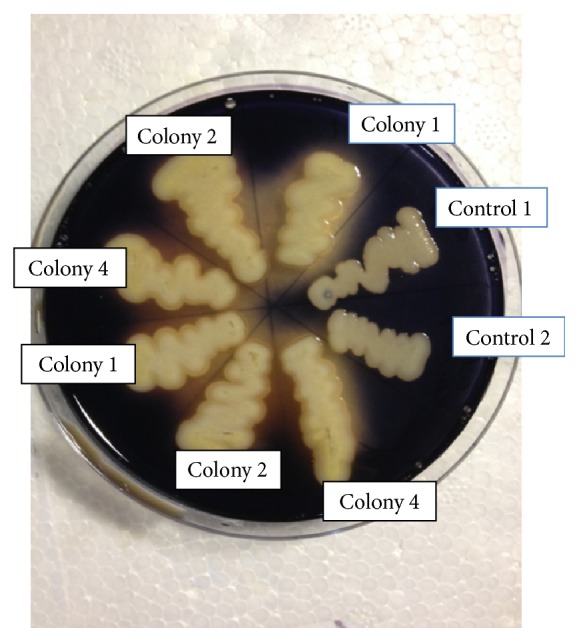
Starch-substrate assay plates showing halo/clear zone by the recombinant Mut^+^ *Pichia* colonies on soluble starch. Colonies 1, 2, and 4 were the recombinant GS115 and indicate the clear halo in plate; controls 1 and 2 did not produce any glucoamylase on the plate.

Linearized recombinant plasmid, pPICZ*α*C_GA, was transformed into* P. pastoris* GS115 by Pichia EasyComp Transformation kit (Invitrogen). This method produces low transformation efficiency with only 8 colonies observed after 4 days of incubation on YPDS agar plate. Five recombinant colonies were selected for colony PCR using AXO1 primers. The amplification results showed that positive recombinants produced two close bands for Mut^+^ in GS115 (2.1 kb and 2.2 kb fragments) using 5′AXO1 and 3′AXO1 primers. Meanwhile the control yeast transformed with pPICZ*α*C produced a 600 bp band. It was observed that recombinant GS115 (Mut^+^) produced recombinant glucoamylase on BMMY plates after 4 days of growth and also produced decolourization zone/clear halo around the* Pichia* colonies with iodine solution compared to control (Figure 2).

**Figure 3:**
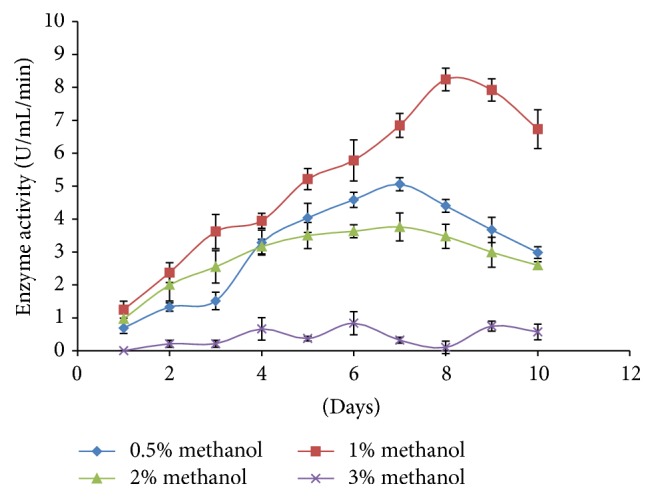
Expression of recombinant glucoamylase (GA) in different concentration of methanol with time.

Heterologous expression of glucoamylase was achieved under the transcriptional control of the AOX1 promoter in* P. pastoris*, and the expression was induced by the supplement of methanol. After eight days of induction, GS115 strain had the highest glucoamylase activity of 8.24 U mL^−1^ at 1% (v/v) methanol (Figure 3), and the recombinant glucoamylase expression level was 0.145 mg mL^−1^ in the culture supernatant. At higher concentration of methanol, glucoamylase expression level was detected to decrease, and no expression was observed when induced with 5% (v/v) of methanol.

### 3.3. Characterization of Recombinant Glucoamylase

#### 3.3.1. Molecular Weight Determination by SDS-PAGE

**Figure 4:**
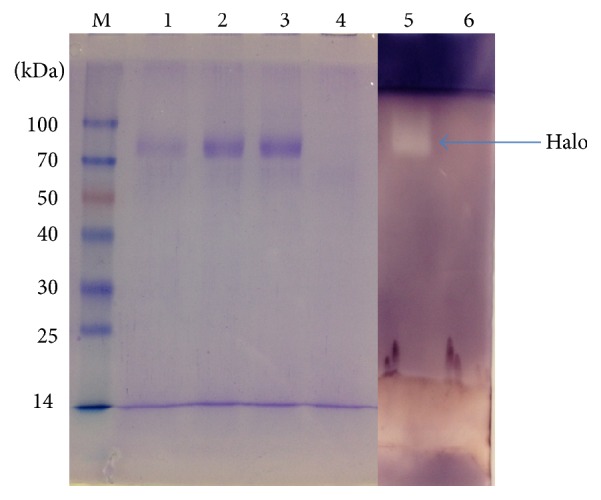
SDS-PAGE and native PAGE analysis of recombinant glucoamylase (GA). Lane 1 and Lane 2 are the 2nd day and 5th day of expression, respectively; Lane 3 indicates the purified recombinant glucoamylase; Lane 4 indicates control at 5th day; Lane 6 also indicates control for activity in native PAGE in 5th day; Lane 5 indicates activity for expressed GA in native PAGE (purified sample).

The molecular weight of the rGA was estimated to be approximately 78 kDa when analyzed on SDS-PAGE (Figure 4). This value was higher than the 55.1 kDa calculated from the deduced amino acid sequences of rGA. The mature rGA had 514 amino acids which was calculated from the Kex2 signal cleavage site to 6x his tag or stop codon of the recombinant plasmid pPICZ*α*C_GA. Based on the results of molecular weight in the SDS-PAGE and the calculated weight of rGA, the expressed recombinant glucoamylase in the culture was monomeric and might be glycosylated. The rGA of* Aspergillus flavus* NSH9 expressed in* Pichia pastoris* was confirmed by four methods via its ability to decolorize or form halo around the* Pichia* colonies after flooding with iodine solution on BMMY plate (Figure 2), zymogram analysis on SDS-PAGE (Figure 4), enzyme assay, and MALDI-TOF/TOF mass spectrometry analysis of peptide after digestion of protein by trypsin (data not shown).

#### 3.3.2. Effects of pH, Temperature, and Kinetics

**Figure 5:**
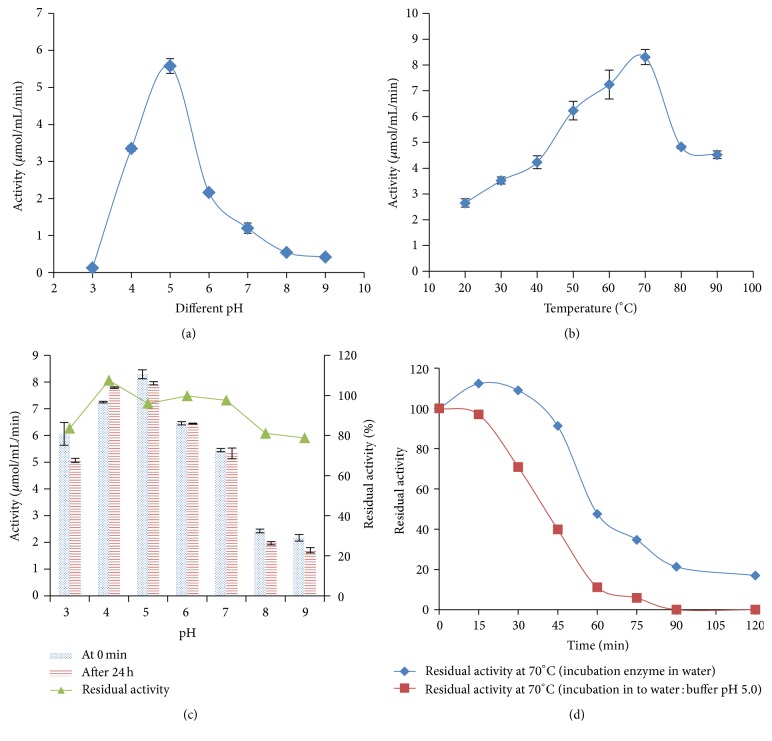
Characterization of the recombinant glucoamylase. (a) Effect of pH on enzyme activity. (b) Effect of temperature on enzyme activity. (c) pH stability of enzyme after 24-hour incubation at 25°C, and the assay condition was at optimum pH (pH 5.0) and temperature (70°C). (d) Thermostability of recombinant glucoamylase at 70°C in two different conditions (diluted enzyme in 0.1 M sodium acetate buffer at pH 5.0 and diluted enzyme in water); assay condition was at optimum pH (pH 5.0) and temperature (70°C). Each value in the panel represents the mean ± SD (*n* = 3).

The pH optimum of rGA for glucoamylase activity was pH 5.0 (Figure 5(a)), and rGA was highly stable over a broad pH range; about 80% of the residual activity was retained after being incubated at pH 3.0 to 9.0 and at 25°C for 24 hours (Figure 5(c)). The optimum temperature of rGA was 70°C, and the value was significantly higher from all values (Figure 5(b)). The rGA was thermally more stable at 70°C, when incubated at neutral pH (water), compared to pH 5.0 with 0.1 M sodium acetate buffer (Figure 5(d)). Approximately, 90% rGA residual activity was observed after 45 min at 70°C at neutral pH and about 40% at pH 5.0 at the same condition. About 90% rGA residual activity was observed after 15 min of incubation at 60°C to 90°C, but at 100°C the rGA residual activity was about 59% after 15 min (Table 1). About 50% rGA residual activity was observed after incubation of 20 min at 90°C and 100°C (Table 1).

The values of Michaelis-Menten constant, *K*_m_ and maximum velocity, and *V*_max_ of the recombinant glucoamylase were determined using Lineweaver-Burk plot and recorded as 5.84 mg mL^−1^ and 153.85 U mg^−1^ protein, respectively, for soluble starch.

#### 3.3.3. Effects of Metal Ions and Different Substrates

Metal ions such as Na^+^, K^+^, Ca^2+^ and Cd^2+^, ions had no significant effect on the recombinant glucoamylase activity at concentration of 1 mM, but higher concentration of 5 mM Na^+^ had negative effect whilst Ca^2+^ had positive effect on enzyme activity (Table 2). The recombinant glucoamylase enzyme was significantly stimulated by Cu^2+^, Fe^2+^, Pb^2+^, Zn^2+^, and Mg^2+^, at 1 mM concentration of ions, but significantly inhibited by Cu^2+^, Pb^2+^, and Zn^2+^ at 5 mM concentration (Table 2). The results revealed that K^+^ and Cd^2+^ had no effect on enzyme activity at different concentrations, and rGA activity was significantly stimulated by only Mg^2+^ ion at different concentration. In this work, it was observed that low concentration of metal ions had a positive effect on enzyme activity in all ions tested. The relative activity of the recombinant glucoamylase was observed on 1% (w/v) of different starches at optimum temperature (70°C) and pH (5.0). The recombinant glucoamylases produced were more active on the breakdown of gelatinized sago starch as compared to soluble starch, amylopectin, and glycogen (Table 3).

## 4. Discussion

The glucoamylase gene from* Aspergillus flavus* NSH9 consisted of two introns and the size of these introns (<100 bp) is typical for most fungal introns [31]. The splice sites of these introns correspond to the fungal consensus sequences, GT – AG boundaries, and internal consensus for lariat formation [32]. From protein BLAST (NCBI), it was determined that this glucoamylase cDNA belonged to the glycoside hydrolases 15 family excluding the starch binding domain (SBD). The single domain structure of glucoamylase (glucoamylase without starch binding domain) was also observed in many fungi such as in* A. niger* [10],* Rhizopus oryzae* [11], and* A. oryzae* [12], which was the similar to the glucoamylase from* A. flavus* NSH9 in this study. Two different forms of glucoamylase, with and without starch binding domain are produced from one gene or two different genes, depending on mRNA splicing [15], site of transcription initiation, posttranslational processing, and limited proteolysis [4]. For example,* A. niger *and* A. awamori *var.* kawachi *produced two GA with and without SBD from the same gene due to proteolytic cleavage of SBD from large GA1 [13], but for* A. oryzae *from two different genes [14]. According to Hata et al. [14], they purified two glucoamylases of* A. oryzae* from solid-state culture (S-GA) and from submerged culture (L-GA). They concluded that the two glucoamylases have different molecular weight; and all of the enzymatic characteristics of the two glucoamylases were similar except the thermal stability and the raw starch digestibility (L-GA could digest the raw starch). The S-GA has a higher amount of carbohydrate than L-GA, and they assumed that the higher glycosylation of S-GA increased its thermal stability and pH stability, similar finding also observed in this study. Basically in the industry, they used the glucoamylase having the starch binding domain like L-GA due to digestibility of raw starch. In this study the glucoamylase that discussed was similar to S-GA from* A. oryzae* having low raw starch digestibility [14]. Glucoamylase is different in the activity on raw and soluble starch based on the structure and domain; glucoamylases with SBD are more active on raw starch compared to single domain glucoamylase like* A. flavus* NSH9 glucoamylase without SBD in this study.

The high degree of similarity of the glucoamylase in this study was observed with the single domain structure of others glucoamylases such as the* A. flavus* NRRL3357 (XP_002375005.1, putative precursor),* A. oryzae* 3.042 (EIT73393.1), and* A. parasiticus* SU1 (KJK64719.1). Meanwhile, the glucoamylase gene of* A. flavus* NSH9 was comparatively less similar to the glucoamylase having starch binding domain such as in* A. awamori* (AEI58995.1),* A. niger* (AIY23067.1), and* A. oryzae* (BAA00841.1).

In the expression study, increasing the concentration of methanol above 1% had negative effect on expression, may be at higher concentrations of methanol causes stress to the cells, decreases cell viability, and facilitates cell lysis and proteolytic degradation [33]. Heterologous expression of glucoamylase in this study was comparatively higher compared to glucoamylases expressed in* Clostridium thermosaccharolyticum* [34],* Sulfolobus solfataricus* [35], and* Thermomyces lanuginosus* [36]. Our finding was in parallel with previous reported studies with molecular weights of GA in fungi to be within a range of 25–112 kDa [9]. Many researchers reported that the molecular weight of recombinant glucoamylase was higher than the estimated molecular weight and it depended on the glycosylation site within the sequence, which supported this study [21, 37–39]. The study did not measure the degree of glycosylation of the recombinant glucoamylase and deglycosylation where this will give a clearer picture of the recombinant enzyme properties and become one of the limitations of this study.

It has been reported that fungal glucoamylases are active at acidic pH [9], and the maximum catalytic activity at pH 5.0 determined in our reinforces many other studies in* A. niger* (pH 4.8) [40],* A*.* tubingensis* (4.5) [37], and* F. solani* (pH 4.5) [41]. The optimum catalytic activity of the recombinant glucoamylase at pH 4.0 to 5.0 expressed in* P. pastoris* was observed by many researchers [21, 38]. A higher optimum temperature of recombinant glucoamylase activity reported in this study was similar to the recombinant glucoamylase from* A*.* tubingensis* expressed in* S. cerevisiae *[37] and from* Rhizomucor pusillus *expressed in* Pichia pastoris* [38]. The rGA from* A. flavus *NSH9 had a higher optimum temperature compared to recombinant glucoamylase from* Chaetomium thermophilum *[21] and* A. awamori* [28]. Meanwhile, pH stability of rGA reported in this study was almost similar to a number of researchers [9, 18, 19, 39, 42]. Glucoamylase that shows high stability over a wide pH range for relatively long period time indicates its suitability for industrial applications [9, 19].

As for the production of glucose in the starch processing industry, it involves two stage processing steps: the liquefaction step at a high temperature (95–105°C) by thermostable alpha amylase and saccharification step by glucoamylase with additional debranching enzymes [43] are normally performed in the industry. Currently, glucoamylases derived from* Aspergillus *sp. are commonly used in starch industry for the saccharification process [39, 43]. But the glucoamylase from fungal sources are active at acidic condition and also less thermostable, and the industrial saccharification takes a long time to produce the desired yield [4], for this it also required pH and temperature adjustments from liquefaction to saccharification steps [39]. The reported recombinant glucoamylase (rGA) from* A. flavus* NSH9 can be used directly after liquefaction process for a complete hydrolysis of the dextrin into *β*-D-glucose, due to its thermostability. Many researchers reported that rapid denaturation of glucoamylase occurred at temperatures above 60°C from* F. solani* [41], and glucoamylase derived from* A. citrinus* [44] was very unstable at temperatures above 55°C. The recombinant glucoamylase from* A. flavus* NSH9 has lower *K*_m_ value compared to* Acremonium *sp. [45] and* R. oryzae *[46], respectively, but higher than* F. solani* [41] and* A. niger* NCIM [47] for soluble starch. Lower *K*_m_ value for starch may be due to a higher number of interactions between the active site of the enzyme and the substrate molecule, resulting in an increased affinity of the enzyme towards the substrate [47]. Our results also observed that low concentration of metal ions exerts positive effect on recombinant enzyme activity. Our results supports the findings reported by Bhatti et al. [41] for some metal such as Mg^2+^ and Ca^2+^ and inhibitory effect of Cu^2+^ and Zn^2+^ at higher concentration as reported by Koç and Metin [19]. There are many methods, which will improve the stability of rGA from* Aspergillus flavus* NSH9, such as immobilization, modification, and protein engineering [48]. Immobilization of enzyme enhances their properties for efficient utilization in industrial process [48]. Magnetically (magnetic nanoparticles, MNPs) immobilized enzymes have advantages for commercial application due to their ease of separation and reusability [48].

## 5. Conclusion

In this work, a novel thermostable glucoamylase cDNA without the substrate binding domain (SBD) of* Aspergillus flavus* NSH9 has been successfully isolated, characterized, and overexpressed in* Pichia pastoris* GS115 producing biologically active recombinant glucoamylase (rGA). These finding opens up for the possibility of upscaling and mass production of the recombinant thermostable glucoamylase in an easier manner. Subsequently, this rGA has the potential for use and application in the saccharification steps, due to its thermostability in the starch processing industries.

## Supplementary Material

Supplementary Figure 1: In the isolation of glucoamylase gene from both the cDNA and genomic DNA, GAAsp_F (5′CGCATGCGGAACAACCTTCTTT 3′) and GAAsp_R (5′CTACCACGACCCAACAGTTGG 3′) primer set successfully amplified the full length of glucoamylase gene which was recorded as 1482 bp from cDNA and 1587 bp from gDNA, respectively. Figure 1 indicated the full length of PCR products of glucoamylase gene from both the cDNA and gDNA.
Supplementary Figure 2: Five motifs: AEPKF, WGRPQRDGP, DLWEEV, ALSNHK and AAELLYDA were found to be highly conserved among fungi glucoamylase. The first 17 amino acids were presumed to be the signal peptide. Thus, the mature glucoamylase1 protein should consist of 476 amino acids with a calculated molecular weight of 50.746 kDa and isoelectric point of 4.76. Five putative asparagine-linked N-glycosylation sites (at 139,198, 255, 369 and 457 position) and another two possible glycosylation site at 120 and 384) were present in the deduced amino acid sequence, which were deduced according to the rule of Asn-X-Thr/Ser. Searches of protein sequences in the NCBI (Protein BLAST) revealed that the sequence of *A. flavus* NSH9 glucoamylase 1 (GA1) showed a high degree of similarity with the glucoamylase sequences from other fungal glucoamylases. Figure 2 indicating the coding sequence of glucoamylase 1(GA1) of both the nucleotides and proteins.

## Figures and Tables

**Table 1:** Thermal stability of recombinant glucoamylase (GA) in different temperature by incubation of diluted enzyme with 0.1 M sodium acetate buffer pH 5.0 (1 : 1; 250 *μ*L diluted enzyme in water : 250 *μ*L 0.1 M sodium acetate buffer pH 5.0).

Time	Residual enzyme activity in different temperature				
	60°C	70°C	80°C	90°C	100°C
0 min	100	100	100	100	100
5 min					83.28
10 min				95.89	77.73
15 min	98.25	96.98	91.53	88.16	58.67
20 min				51.49	49.63
25 min				36.64	33.38
30 min	80.82	70.89	32.8	25.02	20.15
45 min	50.3	39.87	25.32	14.57	12.02
60 min	24.15	11.12	11.0	8.7	0
75 min	11.94	5.82	3.0	0	0
90 min	8.45	0	0	0	0
120 min	0	0	0	0	0

Assay condition was set at optimum pH (5.0) and temperature (70°C). Each value represents the mean of three independent observations.

**Table 2:** Effect of metal ion of recombinant glucoamylase (rGA) activity.

Name of metal	Relative activity of recombinant glucoamylase (rGA)	
	Metal salt (1 mM)	Metal salt (5 mM)
Control (H_2_O)	1.00	1.00
Na^+^	1.04	0.86^*∗*^
K^+^	1.04	0.99
Ca^++^	1.03	1.19^*∗*^
Mg^++^	1.06^*∗*^	1.13^*∗*^
Fe^++^	1.50^*∗*^	0.96
Zn^++^	1.15^*∗*^	0.73^*∗*^
Cu^++^	1.63^*∗*^	0.44^*∗*^
Pb^++^	1.39^*∗*^	0.66^*∗*^
Cd^++^	1.02	1.04

^*∗*^Having significant effect on activity of metal ion (*p* ≤ 0.05 by independent sample *t*-test).

**Table 3:** Relative activity of recombinant glucoamylase on different substrates.

Substrates	Relative activity%
Soluble starch	100
Amylopectin	93.5
Glycogen	33.1
Sago starch	104.7
