# Supplementary material for: Heterologous, Expression, and Characterization of Thermostable Glucoamylase Derived from Aspergillus flavus NSH9 in Pichia pastoris
Source: Biomed Res Int. 2016 Jul 18;2016:5962028. doi: 10.1155/2016/5962028 (PMC4967687; doi:10.1155/2016/5962028)
Supplement: Supplementary file 1 — Supplementary Figure 1: In the isolation of glucoamylase gene from both the cDNA and genomic DNA, GAAsp_F (5′CGCATGCGGAACAACCTTCTTT 3′) and GAAsp_R (5′CTACCACGACCCAACAGTTGG 3′) primer set successfully amplified the full length of glucoamylase gene which was recorded as 1482 bp from cDNA and 1587 bp from gDNA, respectively. Figure 1 indicated the full length of PCR products of glucoamylase gene from both the cDNA and gDNA. Supplementary Figure 2: Five motifs: AEPKF, WGRPQRDGP, DLWEEV, ALSNHK and AAELLYDA were found to be highly conserved among fungi glucoamylase. The first 17 amino acids were presumed to be the signal peptide. Thus, the mature glucoamylase1 protein should consist of 476 amino acids with a calculated molecular weight of 50.746 kDa and isoelectric point of 4.76. Five putative asparagine-linked N-glycosylation sites (at 139,198, 255, 369 and 457 position) and another two possible glycosylation site at 120 and 384) were present in the deduced amino acid sequence, which were deduced according to the rule of Asn-X-Thr/Ser. Searches of protein sequences in the NCBI (Protein BLAST) revealed that the sequence of A. flavus NSH9 glucoamylase 1 (GA1) showed a high degree of similarity with the glucoamylase sequences from other fungal glucoamylases. Figure 2 indicating the coding sequence of glucoamylase 1(GA1) of both the nucleotides and proteins. [file 5962028.f1.docx]

**Supplementary Figures:**

**Suppl. Fig. 1** Full length (1482 bp) of glucoamylase1 (GA1) from cDNA and 1587 bp of PCR product from gDNA; L1 = from cDNA and L2 = from gDNA.

# Suppl. Fig. 2 Coding sequence of glucoamylase derived from *Aspergillus flavus* NSH9. Red color with an underline indicates the signal peptide sequences, yellow shadow are the conserve sequences in fungal glucoamylase, blue color with underline are the potential N glycosylation site of the gene and lowercase character are the intron sequences in the gene.

Suppl. Fig. 1


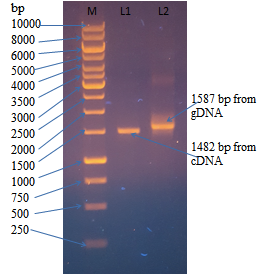


Suppl. Fig. 2

| 1 - ATGCGGAACAACCTTCTTTTTTCCCTCAATGCCATTGCTGGCGCTGTCGCGCATCCGTCCTTCCCTATCCATAAGAGGCAG - 81  1 - **M R N N L L F S L N A I A G A V A** H P S F P I H K R Q - 27  82 - TCGGATCTCAACGCCTTCATTGAGACACAGACACCCATCGCCAAACAGGGCGTCCTCAATAATATCGGCGCTGATGGCAAG - 162  28 - S D L N A F I E T Q T P I A K Q G V L N **N** I **G A D G** K - 54  163 - CTTGTTGAGGGGGCTGCCGCTGGTATCGTTGTAGCCTCCCCATCCAAGAGTAATCCCGACTgttcgtacaatcctaccctc - 243  55 - L V E G A A A G I **V V A S P S**  K S N P D - 74    244 – aagaccgcttgatattaccacagagctaactatatatagACTTCTATACCTGGACGCGCGATGCTGGCCTCACCATGGAAG - 324  55 - Y **F Y T W T R D** A G L T M E - 88  325 - AAGTGATAGAGCAATTCATCGGGGGAGATGCGACTCTCGAGTCCACAATCCAGAATTATGTTGACTCTCAAGCGAAGCAGC - 405  89 - E V I E Q F I G G D A T L E S T I Q N Y V D S Q A K Q - 115  406 - AGGCAGTCTCCAACCCATCAGGCGGCCTGTCGGATGGCTCGGGTCTTGCTGAACCCAAATTTTACGTCAATATCTCTCAAT - 486  116 - Q A V **S N P S G** G L S D G S G L A E P K F Y V **N I S Q** - 142  487 - TCACCGATTCCTGGGGCCGACCCCAGCGCGACGGGCCAGCCTTACGTGCTTCCGCCTTGATCGCATATGGCAACTCTCTGA - 567  143 - F T D S **W G R P Q R D G P A L R A** S A L I A Y G N S L - 169  568 - TTTCCAGCGACAAACAATCTGTTGTCAAAGCTAACATCTGGCCAATTGTCCAGAATGACTTGTCTTATGTGGGTCAATACT - 648  170 - I S S D K Q S V V K A N I W P I V Q N D L S Y V G Q Y - 196  649 - GGAACCAGACCGGGTTTGATCTTTGGGAAGAGGTTCAGGGCAGCTCCTTCTTCACTGTTGCTGTGCAGCACAAAGCCTTGG - 729  197 - W **N Q T G** F **D L W E E V** Q **G S S F F T** V A V Q H K **A L** - 223  730 - TGGAGGGCGATGCGTTTGCAAAGGCACTCGGAGAGGAATGCCAGGCATGCTCCGTGGCGCCTCAAATCCTTTGCCATCTTC - 810  224 - **V E G** D A F A K A L G E E C Q A C S V A P Q I L C H L - 250  811 - AGGACTTCTGGAATGGGTCTGCTGTTCTTTCTAACTTACCAACCAATGGGCGCAGTGGACTGGATACCAACTCTCTTTTGG - 891  244 - Q D F W **N G S A** V L S N L P T N G R S G L D T N S L L - 277  892 - GCTCCATTCACACTTTTGATCCAGCCGCCGCTTGTGATGATACAACATTCCAGCCCTGCTCCTCTCGCGCCCTGTCGAACC - 972  278 - G **S I H T F D P** A A A C D D T **T F Q P C** **S** S R A L S N - 304  973 - ATAAGCTTGTGGTTGACTCTTTCCGGTCGGTCTACGGTATCAACAATGGACGTGGAGCAGGAAAGGCCGCGGCAGTGGGCC - 1053  305 - H K L V **V D S F R** S V Y G I N N G R G A G K A A A V G - 331  1054 – GCTACGCAGAGGACACCTATCAGGGAGGCAATCCATGgttggtactctgtctcatatccaaagcttaaactaatgaatatt – 1134  332 - R Y A E D T Y Q G G N P W - 344  1135 - agGTATCTTACCACCCTGGTCGCTGCGGAATTGCTCTACGACGCCTTGTATCAGTGGGACAAACAAGGTCAAGTGAACGTC - 1215  345 - Y L T T L V A A E L **L Y D A L Y Q W D** K Q G Q V **N V** - 370  1216 – ACTGAAACTTCCCTTCCCTTCTTCAAGGACCTCTCCAGCAATGTCACCACCGGATCCTACGCCAAGTCTTCCTCAGCCTAT - 1296  371 - **T E** T S L P F F K D L S S N V T T G S Y A K S S S A Y - 397  1297 - GAGTCGCTTACGAGCGCTGTCAAGACCTACGCAGACGGCTTCATCTCCGTTGTCCAGGAGTATACTCCCGATGGCGGTGCT - 1377  398 - E S L T S A V K T Y A D G F I S V V Q E Y T P D G G A - 424  1378 - TTGGCTGAGCAGTACAGTCGGGACCAGGGCACCCCAGTTTCGGCATCCGATCTGACTTGGTCTTATGCAGCTTTCTTGAGT - 1458  425 - L A E Q Y S R D Q G T P V S A S **D L T W S Y A A** F L S - 451  1459 - GCTGTTGGACGACGAAACGGCACTGTCCCTGCTAGCTGGGGCTCTTCCACGGCCAACGCAGTTCCAAGCCAATGTTCGGGG - 1539  452 - A V G R R **N G T**  **V P A S W G** S S T A N A V P S Q C S G - 478  1540 - GGTACAGTTTCTGGAAGTTACACTACCCCAACTGTTGGGTCGTGGTAG - 1587  479 - G T V S G S Y T T P T V G S W * - 493 |
| --- |
|  |
